# Artificial intelligence for extracellular vesicle engineering and therapeutic design

**DOI:** 10.20517/evcna.2026.159

**Published:** 2026-09-29

**Authors:** Yunwei Wang, Qingchen Meng, Hui Li, Bonan Zhang, Dan Xing

**Affiliations:** ^1^Third Affiliated Hospital of Zhejiang Chinese Medical University, Hangzhou 310005, Zhejiang, China.; ^2^Arthritis Clinical and Research Center, Peking University People’s Hospital, Beijing 100044, China.; ^3^Arthritis Institute, Peking University, Beijing 100044, China.; ^4^Department of Computer Science, City University of Macau, Macau SAR 999078, China.; ^5^Department of traumatology, orthopedics and disaster surgery, I.M. Sechenov First Moscow State Medical University (Sechenov University), Moscow 119991, Russia.

**Keywords:** Extracellular vesicles, artificial intelligence, AI virtual cells, digital twins, precision nanomedicine

## Abstract

Extracellular vesicles (EVs) are promising platforms for next-generation drug delivery and regenerative therapeutics, yet biological heterogeneity, limited targeting specificity, variable cargo composition, and continued reliance on empirical optimization hinder clinical translation. Artificial intelligence (AI) offers new opportunities to make EV engineering more predictive and data-driven. Building on prior work in AI-assisted EV delivery and emerging virtual-EV modeling, this review presents an evidence-aware, multiscale framework that integrates parental-cell programming, vesicle design, recipient-system modeling, and experimental feedback. At the source level, AI virtual cells (AIVCs) may help predict how perturbations to parental cells influence EV biogenesis and composition. At the vesicle level, AI-guided approaches may support cargo prioritization, surface engineering, and loading optimization. At the recipient level, digital twin models could help simulate EV uptake, intracellular trafficking, microenvironmental interactions, and downstream therapeutic responses. These components may ultimately be integrated with experimental validation into iterative design-test-refinement workflows. Importantly, this review distinguishes direct EV-specific evidence from technologies transferable from adjacent fields and concepts that remain prospective. By defining these evidence boundaries and linking them to key challenges in data quality, model interpretability, manufacturing, and validation, this framework provides a practical roadmap for advancing AI-enabled EV therapeutics from conceptual modeling to testable, translationally relevant engineering strategies.

## INTRODUCTION

Extracellular vesicles (EVs), including exosomes, have emerged as important mediators of intercellular communication and promising candidates for next-generation therapeutics owing to their intrinsic biocompatibility, low immunogenicity, and endogenous targeting capabilities^[1,2]^. Nevertheless, the clinical translation of EV-based therapies remains limited by several persistent challenges, including biological heterogeneity, inconsistent therapeutic performance, limited targeting precision, and difficulties in scalable manufacturing^[3,4]^. At the core of these limitations lies a fundamental issue: as biologically derived nanostructures, EVs have properties that are intrinsically shaped by dynamic parental cell states and are therefore difficult to precisely control using conventional engineering strategies^[5]^. To address these challenges, various engineering approaches have been explored, including genetic modification of parental cells, cargo loading, membrane functionalization, and surface ligand conjugation^[6,7]^. Although these methods can partially enhance EV functionality, most optimization processes still rely heavily on empirical trial-and-error experimentation. Such approaches often fail to capture the highly nonlinear relationships among intracellular regulatory networks, EV biogenesis, cargo sorting, and downstream therapeutic responses.

Recent advances in artificial intelligence (AI), multimodal learning, and systems biology are beginning to reshape this landscape^[8,9]^. By integrating large-scale multi-omics, imaging, and functional datasets, AI-driven frameworks may help shift EV engineering from empirical manipulation toward more predictive and rational design^[10]^. Within this paradigm, EVs are no longer regarded as passive biological byproducts; instead, they can be conceptualized as regulated outputs of dynamic cellular systems^[11]^. A particularly important development in this context is the emergence of AI virtual cells (AIVCs), which could be applied to model how intracellular perturbations influence vesicle biogenesis, cargo composition, and secretion dynamics^[12]^. By integrating transcriptomic, proteomic, metabolomic, and epigenetic information, AIVCs could enable the simulation of parental cell behavior under diverse biological conditions, thereby supporting the rational optimization of EV production at the cellular source level^[13]^.

Among potential parental cell platforms, standardized stem cell-derived or progenitor-like cell sources provide useful examples of how AI-guided modeling may improve EV biomanufacturing. Immunity- and matrix-regulatory cells (IMRCs) derived from human embryonic stem cells have attracted attention because of their relatively standardized differentiation procedures, proliferative capacity, and reduced donor-associated variability^[14]^. These features make IMRCs a representative case for exploring how stable parental-cell states may facilitate computational prediction and closed-loop optimization of EV production. However, IMRCs should be viewed as an illustrative platform rather than a universally optimal EV source, and their broader applicability across disease contexts requires further comparative validation^[15]^.

The predictive capability of AI-guided EV engineering may be further extended by digital twin technologies, which could provide dynamic computational replicas of recipient cells, tissues, or pathological microenvironments^[16]^. By simulating EV uptake, intracellular trafficking, signaling responses, and pharmacodynamic behavior *in silico*, such models may establish a quantitative bridge between upstream EV design and downstream biological function. Integrating these models with AI-guided EV engineering could reduce reliance on empirical screening while improving mechanistic interpretability and translational efficiency.

Together, these developments could support the emergence of a prospective closed-loop EV engineering paradigm in which computational prediction, experimental validation, and iterative optimization continuously interact^[17,18]^. In this adaptive framework, AI models generate hypotheses, automated experimental systems validate therapeutic performance, and newly acquired biological data are subsequently reintegrated to improve predictive accuracy^[19]^. Compared with traditional linear workflows, this strategy may provide a more scalable and systems-oriented route toward precision nanomedicine^[20,21]^.

Several recent reviews have established important foundations for research at the intersection of AI and EV biology. Greenberg *et al*. focused on AI-enabled precision EV drug delivery, emphasizing the use of large-scale EV data to improve targeting and therapeutic delivery^[10]^. Lu *et al*. subsequently provided a broader overview of AI applications in EV engineering, including target identification, selective delivery, drug-delivery optimization, cell-communication analysis, multi-omics integration, and synthetic biology^[22]^. More recently, Liu *et al*. proposed the concept of AI virtual extracellular vesicles (AIVEVs), integrating AIVCs, multi-omics modeling, donor-EV-recipient communication, and a closed-loop workflow linking *in silico* prediction with experimental validation^[23]^. Together, these studies have substantially expanded the conceptual scope of AI-assisted EV research. Accordingly, AIVCs, virtual EV modeling, and closed-loop optimization should not be regarded as concepts introduced solely in the present review.

Against this background, the present review contributes by organizing these emerging technologies within an evidence-aware, multiscale engineering framework rather than introducing another isolated AI application or virtual-EV concept. Specifically, we connect parental-cell programming, cargo and surface engineering, recipient-system modeling, and experimental feedback across the source-vesicle-recipient continuum, while distinguishing EV-validated evidence from transferable technologies and prospective concepts. This perspective emphasizes how computational predictions can be translated into experimentally testable EV designs and how data quality, model interpretability, manufacturing reproducibility, and biological validation constrain this process. Figure 1 summarizes this integrated framework. Table 1 compares conventional and AI-driven EV engineering paradigms, whereas Table 2 summarizes the evidence maturity, supporting evidence, potential value, and key unresolved issues of representative AI-enabled strategies.

**Figure 1:**
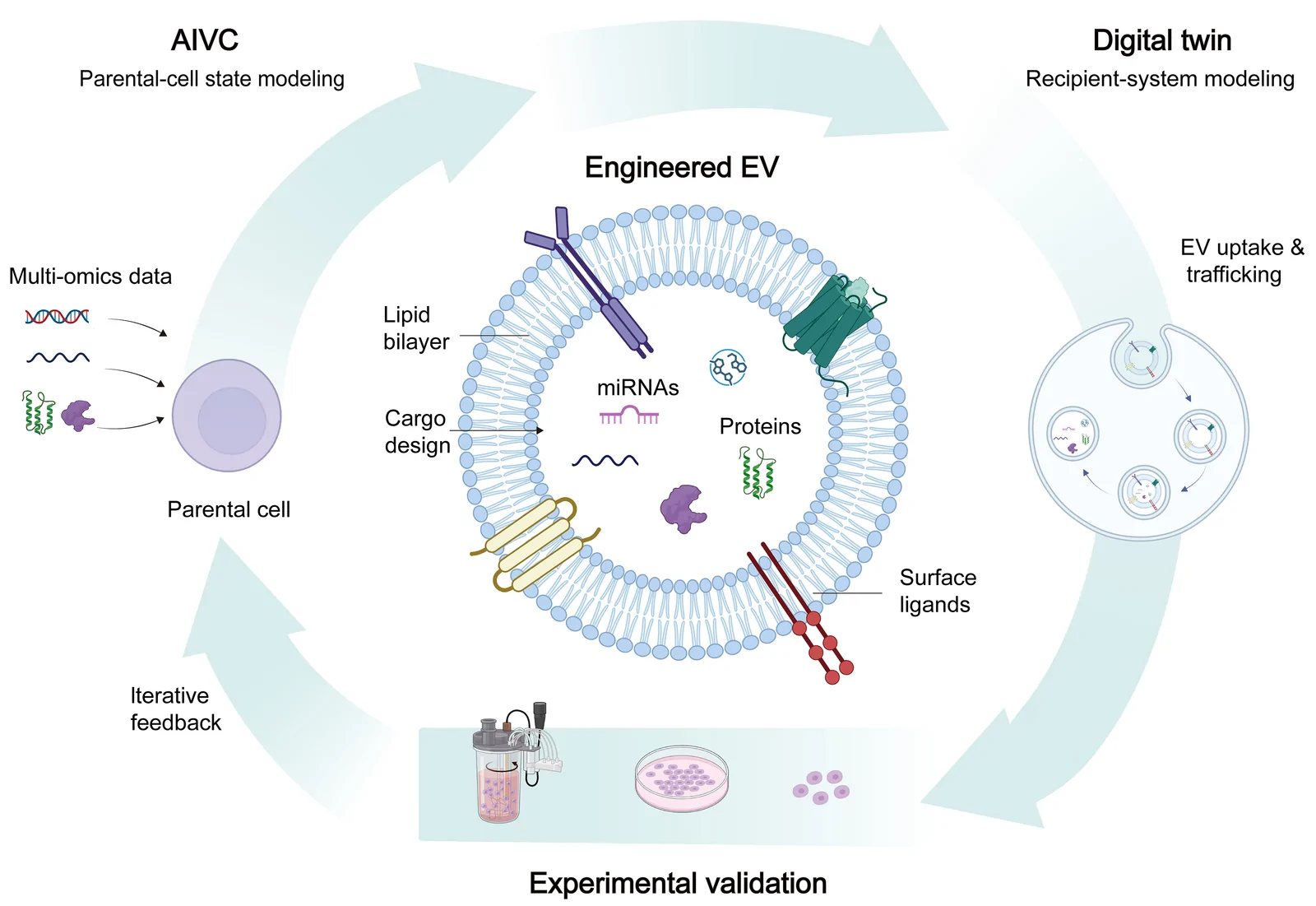
Conceptual framework of AI-driven extracellular vesicle engineering and therapeutic design. The schematic illustrates an integrated workflow in which AIVCs may model parental-cell states and support EV design, engineered EVs may be optimized for cargo composition and surface targeting, digital twin systems may support recipient-system modeling and the simulation of EV uptake and intracellular trafficking, and experimental validation provides feedback for iterative refinement. This framework links source programming, vesicle engineering, *in silico* prediction, and biological validation into a closed-loop strategy that may facilitate precision EV therapeutics. Created in BioRender. AI: Artificial intelligence; EV: extracellular vesicle; AIVC: AI virtual cell; miRNAs: microRNAs.

**Table 1:** Comparison between conventional and AI-driven extracellular vesicle engineering paradigms

**Feature/Dimension**	**Conventional EV engineering**	**AI-Driven EV engineering**
Engineering paradigm	Empirical engineering guided by experimental testing^[5,24]^	Proposed predictive and data-driven engineering guided by emerging AIVC frameworks^[12,22]^
EV design strategy	Modification of selected cargo components or surface features^[7]^	Potential for multidimensional optimization of cargo composition, targeting ligands, and vesicle architecture^[22]^
Optimization workflow	Experimental refinement of cargo loading and surface modification^[5,24]^	Proposed iterative closed-loop optimization integrating computational prediction, experimental validation, and feedback^[23,25]^
Functional evaluation	*In vitro* and *in vivo* functional assessment^[26,27]^	May support complementary *in silico* evaluation through digital twin-based pharmacodynamic modeling^[16,23]^
Mechanistic understanding	Mechanistic interpretation based on experimental observations^[26,27]^	Potential for dynamic prediction of multiscale biological interactions and cellular responses, requiring experimental validation^[12,28]^
Manufacturing and scalability	Susceptible to variability arising from biological heterogeneity^[26]^	May improve standardization and scalability through computationally optimized engineering parameters^[22]^
Data integration	Often limited by fragmented datasets and incomplete data integration^[29]^	Potential for integration of heterogeneous single-cell multi-omics data^[9]^
Translational potential	Challenges in reproducibility and cross-study generalization^[27]^	Potential for enhanced precision, adaptability, and translational predictability for personalized nanomedicine^[10]^

AI: Artificial intelligence; EV: extracellular vesicle; AIVC: AI virtual cell.

**Table 2:** Current evidence maturity of AI-enabled strategies for EV engineering

**Approach**	**Evidence maturity**	**Representative evidence**	**Potential value**	**Key unresolved issue**
AIVC-guided source programming	Transferable	Virtual-cell and perturbation models^[12,28]^	Source-level EV optimization	Limited EV-specific validation
AI-guided cargo design	Prospective	Network modeling and EV cargo studies^[30,31]^	Rational cargo prioritization	Limited causal data
AI-assisted surface engineering	Emerging	Protein design and engineered EV targeting^[32-34]^	Improved targeting specificity	Uncertain performance on EV membranes
Predictive cargo-loading modeling	Transferable	EV cargo-sorting mechanisms^[35-37]^	Improved loading efficiency	Incomplete sorting rules
EV digital twins	Prospective	Medical digital twins and AIVEV frameworks^[16,23,38]^	*In silico* response prediction	Limited EV-specific validation
Closed-loop AI optimization	Prospective	AIVEV, automated experimentation, and active learning^[23,25,39]^	Iterative design refinement	Lack of standardized feedback data

AI: Artificial intelligence; EV: extracellular vesicle; AIVC: AI virtual cell; AIVEV: AI virtual extracellular vesicle.

## AIVCS FOR EV SOURCE PROGRAMMING

### Concept of AIVCs in EV research

The functional heterogeneity of EVs is strongly influenced by the dynamic biological states of their parental cells^[40]^. EV biogenesis is tightly regulated by coordinated intracellular processes, including gene expression programs, epigenetic remodeling, metabolic adaptation, organelle communication, and vesicular trafficking^[41,42]^. Together, these processes shape the molecular composition, membrane architecture, biological activity, and therapeutic potential of secreted vesicles^[43]^. EV populations generated under different physiological or pathological conditions often exhibit substantial variability in protein cargo, nucleic acid content, lipid composition, and immunomodulatory capacity^[44,45]^.

Conventional EV engineering strategies have primarily focused on post-isolation modification approaches, including electroporation-mediated cargo loading, chemical conjugation, and membrane surface functionalization^[24,46,47]^. Although these methods provide partial control over vesicle composition, they frequently suffer from limited loading efficiency, structural instability, cargo leakage, and compromised vesicle integrity^[29,48]^. More importantly, such strategies do not fundamentally address the intrinsic biological variability originating from differences in parental cell states during EV production. As a result, batch-to-batch inconsistency remains a major obstacle to the translational development and industrial scalability of EV therapeutics^[49]^. Increasing evidence suggests that more effective control of EV function may require direct regulation of the cellular systems responsible for vesicle biogenesis.

In this context, AI-enabled virtual cell models provide a potential framework for exploring and predicting the intracellular determinants of EV production. Rather than analyzing EVs as isolated extracellular particles, AIVCs could model vesicle secretion as an integrated systems-level output of cellular physiology. AIVCs are computational models that integrate multidimensional biological information into unified representations of cellular states. By combining deep learning, graph neural networks (GNNs), probabilistic inference, and systems biology approaches, these models capture highly nonlinear relationships across genomic, transcriptomic, proteomic, metabolomic, and epigenomic layers. Compared with reductionist strategies focused on isolated signaling pathways or individual molecular targets, AIVCs could provide a more comprehensive understanding of how intracellular regulatory networks influence EV biogenesis and cargo selection^[12,13]^. Within this framework, EVs can be regarded as programmable biological outputs whose molecular composition and functional properties dynamically reflect the parental cell's regulatory state. This conceptual shift provides an important foundation for predictive EV engineering. Rather than relying solely on repeated empirical optimization, virtual-cell models could explore how specific intracellular perturbations may affect downstream EV phenotypes. This approach may support a gradual shift from descriptive characterization toward more rationally guided EV design.

### Multi-omics integration and network architecture

A key advantage of AIVCs lies in their ability to integrate highly heterogeneous biological datasets into coherent and predictive models of cellular function. Modern cellular systems generate large volumes of high-dimensional data from single-cell RNA sequencing (scRNA-seq), chromatin accessibility profiling, spatial transcriptomics, quantitative proteomics, phosphoproteomics, metabolomics, lipidomics, and EV-associated small RNA sequencing^[50,51]^. Individually, each dataset captures only a limited aspect of cellular regulation. However, EV biogenesis emerges from coordinated interactions among these regulatory layers across multiple biological scales. Critically, the reliability of such models depends on high-quality, standardized, and functionally annotated multi-omics datasets, as technical variability in EV isolation, sequencing, normalization, and annotation can propagate into downstream computational predictions^[26,29]^.

To address this complexity, AIVCs could employ multi-omics integration frameworks that embed diverse datasets into shared latent feature spaces. Transcriptomic profiles derived from parental cells can therefore be computationally linked with EV proteomic and RNA cargo datasets, enabling reconstruction of intracellular-to-extracellular information transfer pathways. Through this integrative strategy, AI models may identify latent regulatory relationships that conventional statistical analyses often miss. However, although current AIVC frameworks may capture broad regulatory trends, they remain limited in accurately modeling stochastic cargo loading at single-vesicle resolution, which continues to constrain predictive precision in EV engineering.

GNNs are particularly well suited for modeling such systems because intracellular regulation is inherently organized as interconnected molecular networks rather than linear signaling cascades^[52]^. Genes, proteins, metabolites, transcription factors, and signaling intermediates can be represented as nodes in graph architectures, while their regulatory interactions can be represented as weighted edges. By training on large-scale biological datasets, GNN-based AIVCs could be used to infer candidate relationships between intracellular signaling dynamics and EV cargo selection mechanisms^[30,53]^. Systems-level network modeling further enables identification of critical molecular hubs involved in EV biogenesis^[54,55]^.

For example, AI models could reconstruct miRNA-mRNA regulatory circuits associated with EV RNA sorting, thereby linking intracellular transcriptional programs with EV composition. Similarly, AIVCs may help predict how metabolic stress influences endosomal trafficking pathways, membrane lipid remodeling, and vesicle secretion dynamics. These applications illustrate a prospective route toward quantitatively predictive EV modeling. However, direct EV-specific validation remains limited.

### Modeling EV biogenesis as a systems-level process

EV biogenesis is governed by highly interconnected molecular pathways and is strongly influenced by cellular context, making it difficult to fully dissect using conventional experimental approaches alone^[35,56]^. Vesicle formation involves coordinated interactions among endosomal sorting complexes required for transport (ESCRT), Rab GTPase-mediated trafficking systems, lipid raft dynamics, cytoskeletal remodeling, autophagic pathways, and intracellular stress responses^[57]^. Because these processes operate simultaneously across multiple spatial and temporal scales, perturbation of a single regulatory component frequently produces indirect effects across several interconnected pathways^[58]^.

AIVCs could help address this challenge by modeling EV biogenesis and cargo sorting as emergent properties of dynamic cellular systems rather than as isolated molecular events. Such systems-level modeling may be particularly useful when examining specialized parental cell populations with distinct functional properties.

Specialized parental cell populations can serve as useful model systems for examining how AIVCs may link intracellular regulatory states with EV biogenesis and cargo composition. For example, human embryonic stem cell-derived IMRCs have been reported to display relatively standardized differentiation features, proliferative capacity, immunomodulatory activity, and reduced donor-associated variability compared with some primary mesenchymal cell sources^[14]^. These properties may improve computational tractability because more stable parental-cell states can facilitate modeling of EV production across batches.

In this context, AIVCs could explore how IMRC-associated regulatory programs influence vesicle biogenesis, cargo sorting, and functional output. For instance, computational models may evaluate how mesenchymal and immunoregulatory gene networks affect intracellular trafficking dynamics and the enrichment of anti-inflammatory RNAs, matrix-regulatory proteins, or anti-fibrotic mediators in secreted EV populations. Importantly, this example illustrates a broader principle: AI-guided source programming could be extended to different parental-cell systems when sufficiently standardized molecular, functional, and manufacturing datasets are available. Therefore, IMRCs should be positioned as one representative model for AIVC-assisted EV source optimization rather than as the central focus of AI-driven EV engineering.

### *In silico* perturbation for EV optimization

The predictive value of AIVCs is particularly relevant to *in silico* perturbation analysis. Machine-learning models have demonstrated the ability to predict cellular responses to defined perturbations in single-cell systems^[28]^. These approaches may therefore provide transferable computational strategies for EV source programming. In EV applications, virtual-cell models could prioritize candidate genetic, pharmacological, metabolic, or environmental perturbations for subsequent experimental validation, thereby reducing the initial experimental search space. One potential application would involve simulating interferon-gamma (IFN-γ) stimulation in IMRCs. Experimental studies have shown that IFN-γ induces stronger indoleamine 2,3-dioxygenase 1 (IDO1) upregulation in IMRCs than in primary umbilical cord-derived mesenchymal stem cells (UCMSCs)^[14]^. Because IDO1 is an important immunoregulatory component of the tryptophan-kynurenine pathway, this differential response provides a biologically relevant perturbation for investigating whether parental-cell immune states are reflected in EV composition and function.

Through computational perturbation analysis, AIVCs could be used to explore cytokine dose, exposure duration, and treatment timing as candidate variables influencing EV composition and secretion. Model outputs could include predicted changes in immunoregulatory RNA or protein signatures, metabolite profiles, and EV production characteristics, all of which would require direct EV-level validation. AI models could also evaluate potential trade-offs involving cellular stress, metabolic burden, and vesicle yield. Together, these analyses would provide a forward-design framework for prioritizing parental-cell conditioning strategies for experimental testing.

### Toward programmable EV-producing cell factories

Integration of AIVCs with scalable parental-cell systems could support the future development of programmable EV-producing cell factories. In principle, this strategy is not restricted to a single cell source. Any parental-cell platform with stable molecular profiles, controllable differentiation or expansion procedures, reproducible EV output, and well-annotated functional datasets could be incorporated into an AIVC-guided optimization workflow. Within such a framework, computational models could be used to evaluate how culture conditions, genetic modulation, cytokine stimulation, metabolic state, or biophysical parameters may influence EV secretion rate, vesicle size distribution, cargo composition, and functional potency [Figure 2].

**Figure 2:**
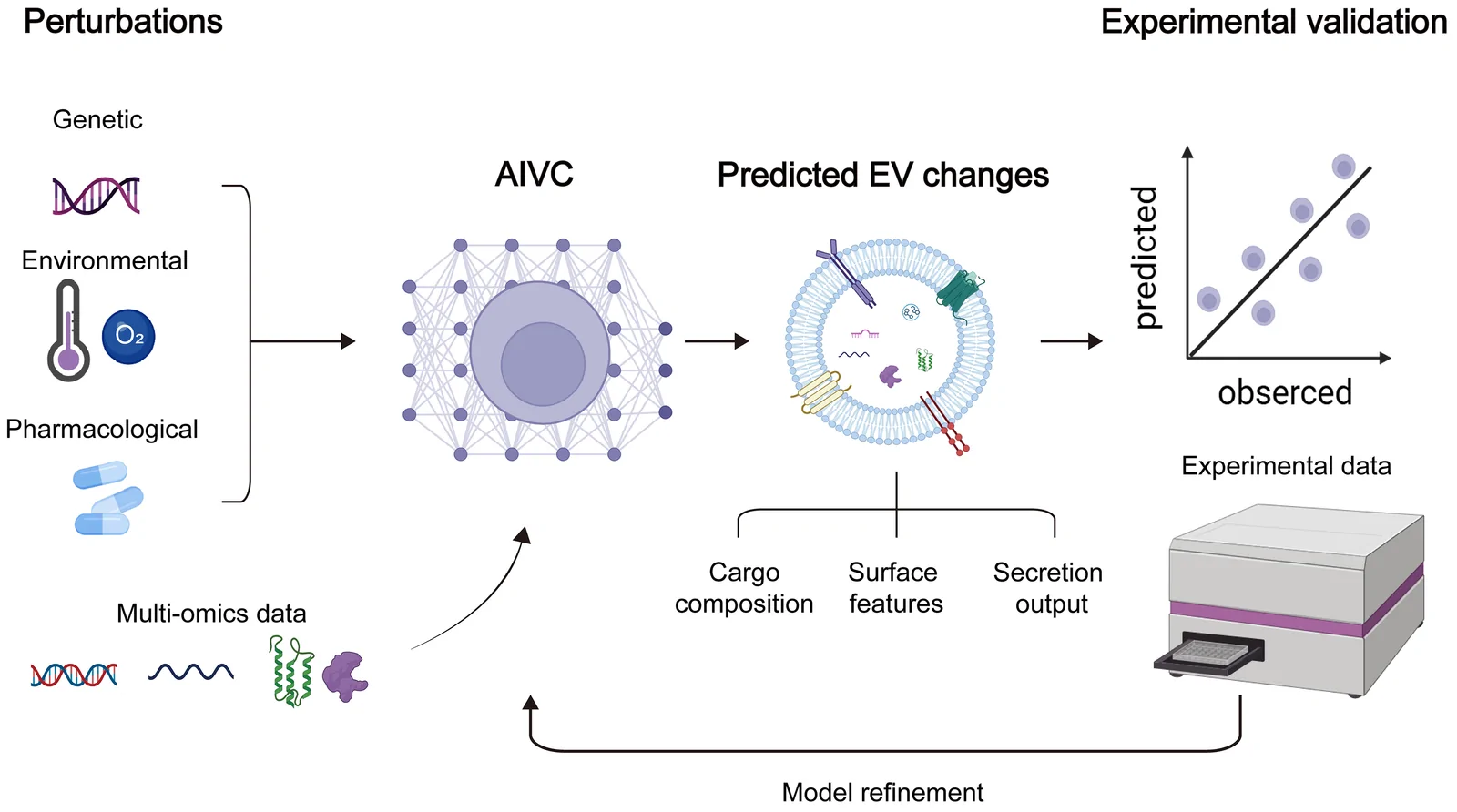
AIVC-guided source programming for optimized EV production. This diagram shows how multi-omics datasets from parental cells, including genomic, transcriptomic, proteomic, and metabolic information, could be integrated into AIVC models to predict potential EV changes in response to defined perturbations. AIVC models may use *in silico* perturbations, such as genetic modulation, environmental changes, or small-molecule treatment, to estimate changes in EV cargo composition, surface features, and secretion output. The predicted outputs can then be compared with experimental data to support model refinement and may help inform subsequent source-cell perturbation and EV production strategies. Created in BioRender. AIVC: Artificial intelligence virtual cell; EV: extracellular vesicle.

IMRCs provide one illustrative example of how a relatively standardized parental-cell platform may be integrated into this strategy. Their defined derivation procedures and reduced donor-associated variability may support more reproducible modeling of parental-cell states and EV production. For example, AI models could reconstruct intracellular synthesis, trafficking, and secretion pathways associated with selected functional mediators, such as matrix-regulatory or immunomodulatory molecules, and then identify regulatory bottlenecks that influence their enrichment in secreted vesicles. Similarly, biophysical parameters, including cell size, membrane dynamics, and vesicle budding behavior, could be incorporated into predictive models to optimize both biochemical cargo and physical vesicle properties.

More broadly, programmable EV-producing cell factories should be understood as a generalizable design concept rather than an IMRC-specific strategy. AIVC-guided optimization may be applied to mesenchymal stromal cells, induced pluripotent stem cell-derived cells, immune cells, epithelial cells, or engineered producer cell lines, depending on the therapeutic goal and manufacturing requirements. Future studies should therefore compare different parental-cell platforms under standardized experimental and computational conditions to determine which sources best suit specific EV therapeutic applications. This broader framing keeps the focus on AI-driven source programming while using IMRCs only as a representative case model.

### AI-GUIDED ENGINEERING OF EV COMPOSITION AND TARGETING

EV therapeutic performance depends primarily on two interrelated determinants: the molecular composition of their internal cargo and their ability to selectively interact with target cells. As naturally secreted nanoscale carriers, EVs transport proteins, nucleic acids, lipids, metabolites, and signaling complexes that collectively regulate inflammation, immune modulation, extracellular matrix remodeling, angiogenesis, and tissue repair. However, native EVs are often constrained by considerable heterogeneity, limited controllability, and insufficient targeting specificity, which restrict their utility in precision therapy^[49,57]^.

Traditional EV engineering has largely relied on post-isolation modification strategies, including electroporation-mediated cargo loading, sonication-assisted incorporation, membrane extrusion, and chemical conjugation of targeting ligands. Although these methods can improve certain functional properties, they often introduce membrane damage, reduce cargo retention, compromise vesicle stability, or interfere with endogenous biological activity^[24]^. Moreover, such approaches usually optimize individual parameters in isolation and therefore fail to account for the coordinated relationships among EV cargo composition, membrane architecture, targeting behavior, and intracellular trafficking.

By contrast, AI-guided EV engineering introduces a more integrated and predictive framework. By integrating computational modeling, systems biology, and large-scale biological datasets, AI may support more rational optimization of EV cargo composition and surface properties according to predefined therapeutic objectives. Compared with conventional empirical modification strategies, this approach may improve design efficiency and functional predictability while better accounting for the coordinated relationships among EV structural and biological features. This shift marks an important transition from empirical vesicle manipulation toward coordinated and predictive engineering of EV performance.

### Rational design and optimization of EV cargo

EV cargo is highly complex and includes miRNAs, mRNAs, long non-coding RNAs, proteins, lipids, metabolites, and regulatory intermediates. These molecules do not act independently. Rather, they form interconnected networks that cooperate to shape cellular responses in recipient cells. As a result, the biological activity of EVs is determined not by a single molecule, but by the combined effects of multiple cargo components acting across different signaling layers^[31]^. For example, EV-associated miRNAs may suppress inflammatory transcription factors, while co-packaged proteins regulate matrix remodeling or metabolic adaptation^[59]^. Lipid components can further influence membrane fusion, vesicle stability, and intracellular trafficking efficiency, thereby indirectly affecting cargo delivery. Understanding EV function therefore requires a systems-level perspective that captures the coordinated behavior of these multilayered molecular modules.

AI-based modeling may provide a useful strategy for reconstructing such regulatory architectures. By integrating transcriptomic, proteomic, lipidomic, metabolomic, and small RNA sequencing data from parental cells and EVs, computational models may identify key molecular hubs linked to defined biological phenotypes. GNNs, probabilistic inference models, and attention-based deep learning architectures are particularly useful in this setting because they can model nonlinear and hierarchical interactions among heterogeneous biological variables. Through these approaches, AI systems may help infer how specific cargo combinations influence downstream outcomes, including inflammatory signaling, senescence, fibrosis, proliferation, angiogenesis, and immune activation. Rather than focusing on individual therapeutic molecules, researchers could evaluate how coordinated cargo modules reshape signaling landscapes in recipient cells. These approaches may also help identify EV cargo patterns associated with specific biological functions. For example, vesicles enriched in anti-inflammatory miRNAs, matrix-regulatory proteins, or metabolic regulators may exhibit distinct immunomodulatory or tissue-reparative properties under defined pathological conditions. However, EV-specific experimental validation remains necessary to establish causal relationships between computationally identified cargo patterns and biological outcomes.

Building on network-based cargo analysis, AI could further support the forward design of EV cargo architectures for specific therapeutic objectives. In conventional workflows, researchers first select cargo molecules empirically and then evaluate them experimentally. However, biological systems rarely respond linearly to single-factor interventions. AI-guided forward design reverses this process by starting from the desired biological outcome. For instance, a therapeutic objective may involve suppressing nuclear factor-κB (NF-κB) signaling, enhancing chondrocyte matrix synthesis, inhibiting macrophage M1 polarization, or promoting angiogenic repair. Computational models could then prioritize candidate combinations of RNAs, proteins, and metabolites predicted to drive these responses in a coordinated manner.

This strategy relies on iteratively simulating signaling responses under different cargo configurations. Machine-learning algorithms could evaluate how specific molecular combinations affect pathway activation thresholds, feedback regulation, and network robustness. In this way, AI systems may help prioritize cargo modules predicted to produce synergistic effects relative to individual therapeutic components. Predicted cargo profiles may subsequently be implemented through parental cell programming, synthetic biology-based modification, or selective loading strategies. AI-guided modeling may also help evaluate the compatibility among cargo components. Some molecules may interfere with one another during intracellular trafficking or vesicle packaging, whereas others may stabilize shared regulatory networks. By accounting for these relationships, computational optimization may improve the predicted functional and structural compatibility of candidate cargo combinations. As a result, EV engineering evolves from additive modification toward coordinated systems-level design, in which the cargo landscape is configured as an integrated therapeutic module.

### AI-driven surface engineering for precision targeting

In addition to cargo composition, the ability of EVs to selectively reach and interact with target cells is a major determinant of therapeutic efficacy. Native EVs naturally employ membrane proteins, lipids, glycans, and adhesion molecules to mediate cellular recognition and uptake. However, these endogenous targeting mechanisms are often nonspecific and insufficiently efficient *in vivo*, particularly because of systemic circulation, immune clearance, extracellular matrix barriers, and interactions with off-target tissue. AI-guided surface engineering provides a systematic approach to improving targeting performance. Recent advances in generative protein modeling, structure prediction, and sequence optimization have enabled the design and prioritization of high-affinity protein binders in adjacent fields^[32]^. These approaches could be adapted to prioritize candidate targeting motifs against inflammatory endothelial markers, fibrosis-associated extracellular matrix proteins, or tumor-associated receptors based on predicted affinity and structural compatibility, although their performance on EV membranes would require experimental validation.

Importantly, targeting elements can be tailored not only to cell type but also to disease state. In inflammatory disorders, EVs may be engineered to preferentially target activated macrophages or inflamed vascular endothelium expressing disease-associated adhesion molecules such as intercellular adhesion molecule 1 (ICAM-1)^[33,60]^. In regenerative settings, surface ligands may be used to improve EV accumulation or retention within injured tissues by targeting extracellular-matrix components such as collagen^[34]^. Such surface-engineering strategies may enhance local EV delivery and potentially reduce nonspecific uptake or off-target distribution, although their effects on systemic circulation and clearance depend on the specific EV formulation and surface modification^[61]^.

Beyond designing individual ligands, AI may also help optimize the EV surface as an integrated structural interface. EV-cell interactions are influenced not only by ligand identity, but also by ligand density, spatial arrangement, membrane fluidity, glycosylation status, lipid composition, and nanoscale surface topology. These properties collectively influence how EVs interact with biological barriers, circulate in the bloodstream, adhere to target tissues, and undergo cellular internalization^[57]^. Variations in ligand density or retention-enhancing surface modifications may introduce trade-offs among targeting efficiency, immune interactions, cellular uptake, and cargo release, thereby requiring empirical optimization. These trade-offs make surface optimization a multidimensional design problem rather than a single-parameter adjustment.

AI-based modeling could support simultaneous evaluation of these competing variables. By integrating molecular dynamics simulations, membrane interaction analyses, and biodistribution data, computational frameworks may help predict how changes in EV surface architecture influence *in vivo* behavior. Such modeling could support a more rational balance among circulation stability, targeting specificity, uptake efficiency, and intracellular delivery performance. In practical terms, engineered EVs could therefore be designed not only to recognize target cells more selectively, but also to enter them efficiently and deliver their cargo in a functionally meaningful manner.

### Predictive modeling of cargo loading mechanisms

A further challenge in EV engineering lies in understanding and controlling cargo loading. Efficient incorporation of therapeutic molecules into EVs depends on their compatibility with endogenous sorting machinery, including ESCRT-associated pathways, RNA-binding proteins, lipid raft dynamics, and vesicular trafficking systems. Not all candidate cargo molecules are equally suitable for vesicular packaging. Some RNAs may show poor enrichment, whereas some proteins may disrupt vesicle formation or compromise membrane stability. Increasing intracellular expression alone does not necessarily ensure efficient EV cargo loading^[35]^. Instead, the molecular determinants of selective packaging must be considered directly. AI-guided predictive modeling may provide a complementary approach by linking structural and sequence features of candidate molecules to their likelihood of EV incorporation.

Machine-learning systems could analyze nucleotide motifs, protein domains, post-translational modifications, charge distributions, and intracellular localization patterns associated with efficient sorting. In parallel, computational models could evaluate interactions between candidate RNA cargo molecules and experimentally established RNA-sorting proteins such as hnRNPA2B1 and YBX1^[36,37]^. By identifying molecular features associated with successful loading, AI systems may help derive design principles for improving cargo compatibility with EV biogenesis pathways. Such predictions could guide rational modification of therapeutic molecules and prioritize candidates for experimental validation.

### Unified AI frameworks for coordinated EV engineering

One potential strength of AI-guided EV engineering is the ability to integrate cargo optimization, targeting design, and loading efficiency within a single computational framework. Conventional engineering strategies often treat these variables separately, resulting in fragmented optimization and limited translational performance. In biological systems, however, these parameters are deeply interconnected^[35,57]^. For example, changes in membrane composition may alter intracellular sorting behavior, while certain cargo molecules may influence vesicle stability or affect surface ligand presentation. Targeting requirements may also constrain vesicle size, membrane rigidity, or circulation half-life. Optimization of one property in isolation may therefore compromise others^[62]^. AI frameworks could address this limitation by simultaneously modeling these multidimensional relationships^[63]^. Through systems-level design, engineered EVs could be considered as integrated biological entities in which cargo composition, surface architecture, stability, and targeting behavior are jointly optimized [Figure 3]. Such integration may better reflect the complexity of biological environments and support translational development.

**Figure 3:**
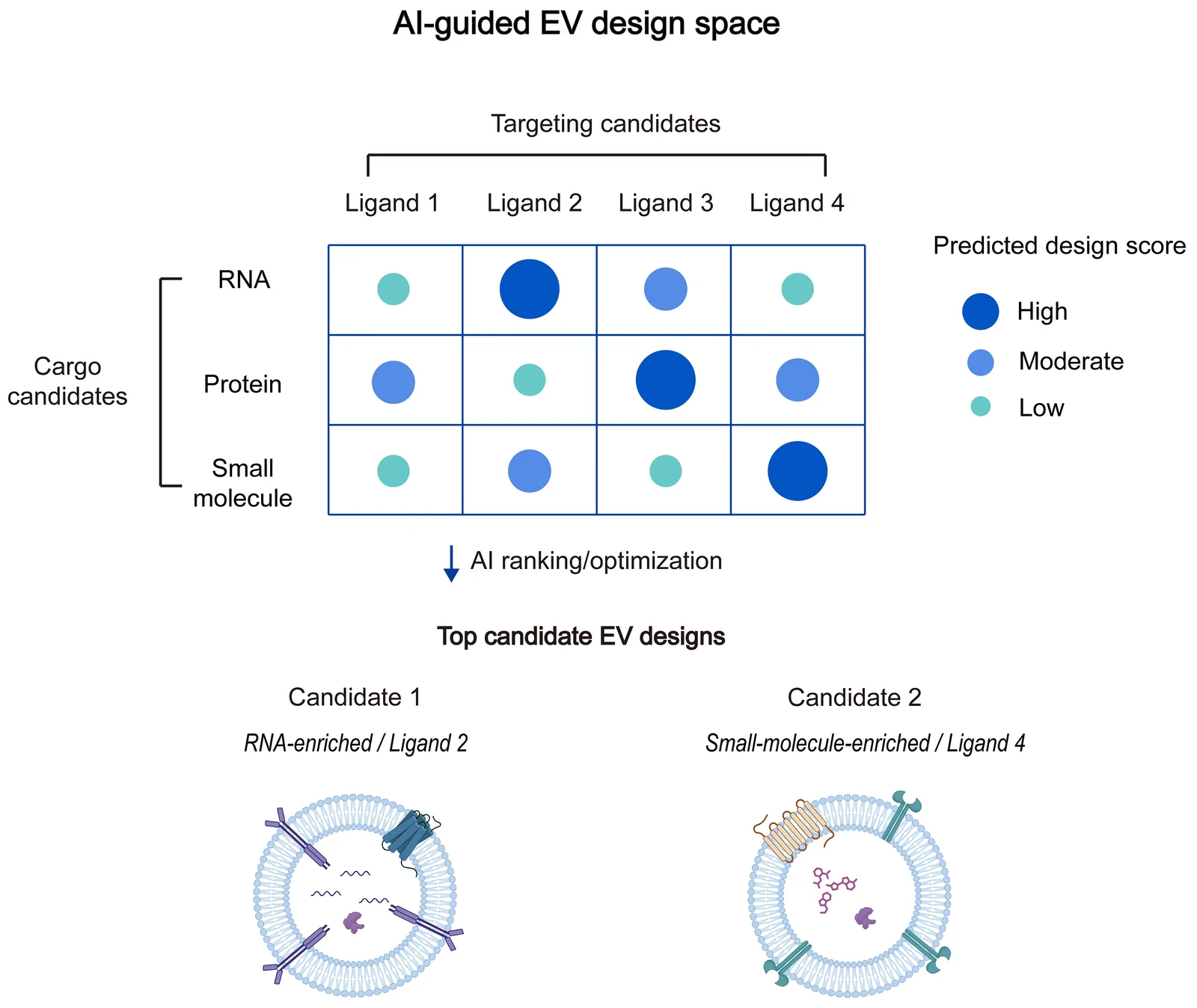
AI-guided coordinated engineering of EV cargo composition and surface targeting. The schematic summarizes a prospective AI design framework for coordinated EV engineering. Candidate cargo types, including RNA, proteins, and small molecules, could be evaluated in combination with candidate surface ligands to define an EV design space and generate predicted design scores. AI-based ranking and optimization may then help prioritize cargo-ligand combinations with potentially favorable design characteristics. These computationally prioritized EV designs could serve as candidates for subsequent experimental validation and further refinement of cargo loading and targeting performance. Created in BioRender. AI: Artificial intelligence; EV: extracellular vesicle.

Integrating digital twin approaches could add an *in silico* layer for evaluating candidate EV designs before experimental testing. Such models may eventually support the assessment of biodistribution, cellular uptake, intracellular trafficking, and off-target effects under different biological conditions. By combining cargo optimization, surface engineering, and digital twin-based pharmacological evaluation, AI-guided EV engineering may provide a more comprehensive approach to precision nanomedicine^[23]^.

## DIGITAL TWINS FOR EV-TARGET CELL INTERACTIONS AND PHARMACODYNAMICS

The therapeutic efficacy of engineered EVs depends not only on their molecular design but also on their dynamic interactions with recipient cells in complex biological environments. Although AI-guided engineering approaches may support the rational optimization of EV cargo composition, membrane architecture, and targeting specificity, predicting how these vesicles behave *in vivo* remains a major challenge. Conventional experimental systems, including two-dimensional culture models, static co-culture assays, and animal studies, provide important information but often fail to capture the multiscale, time-dependent, and context-sensitive nature of EV-mediated communication.

Digital twin approaches may provide a framework for linking engineered EV properties with downstream biological behavior. However, EV-specific digital twin applications remain at an early conceptual stage, with recent studies primarily proposing virtual EV or predictive modeling frameworks rather than experimentally validated, patient-specific digital twins^[23]^. Accordingly, these systems should currently be regarded as emerging predictive models rather than mature clinical digital twins.

By integrating molecular profiles, cellular signaling states, tissue microenvironmental parameters, and biophysical transport dynamics, digital twin models could simulate EV uptake, intracellular trafficking, signaling responses, and downstream pharmacodynamic behavior [Figure 4]. Such multiscale modeling could link upstream AI-guided EV engineering with downstream biological function, allowing candidate EV designs to be evaluated computationally before experimental validation. Therefore, context-aware digital twin models may support more rational preclinical optimization.

**Figure 4:**
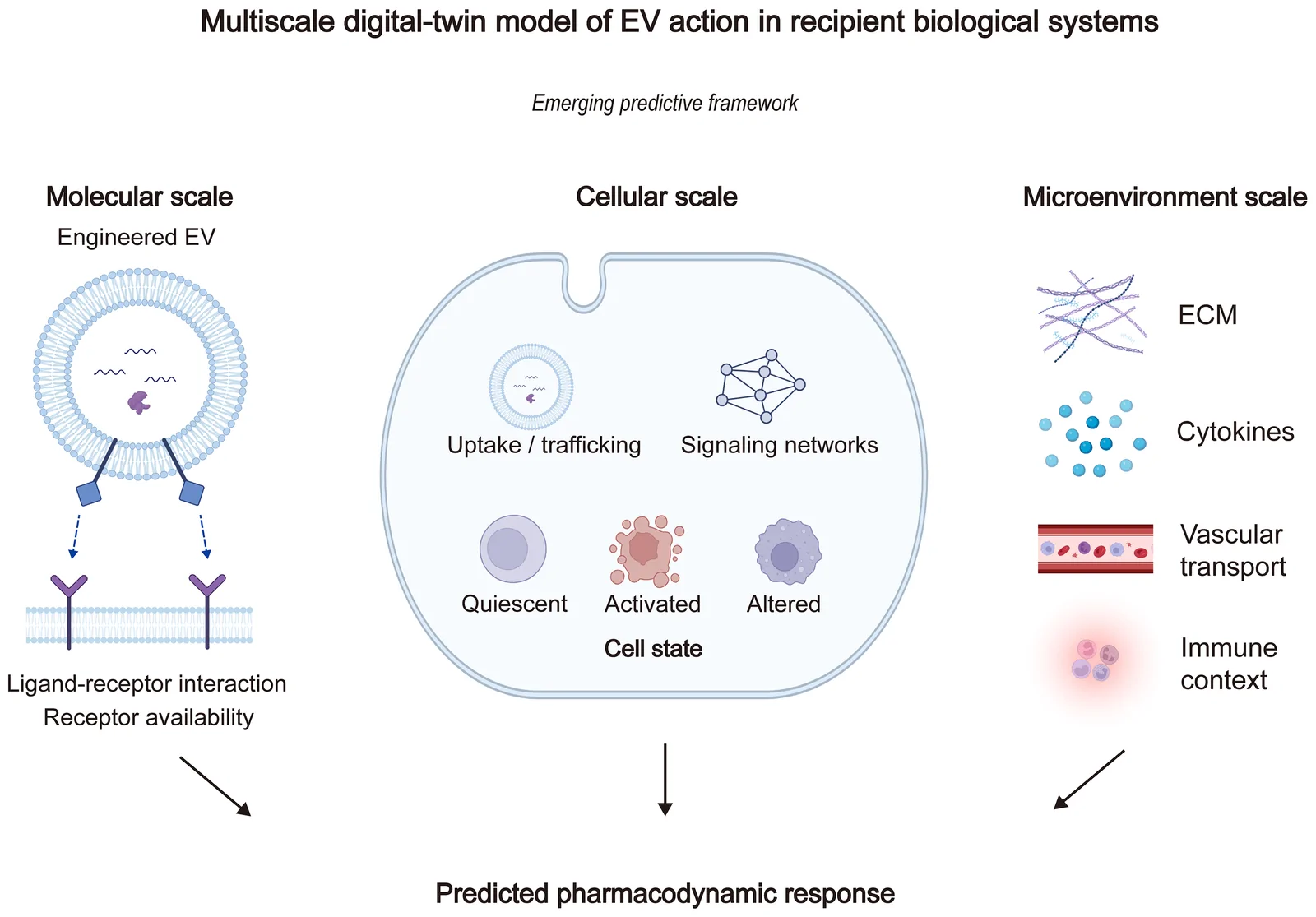
Digital twin modeling of EV-target cell interactions and virtual pharmacodynamics. This schematic illustrates how digital twin systems may represent the multiscale behavior of engineered EVs after administration. Such models could incorporate EV-target cell recognition, ligand-receptor binding, receptor availability, endocytosis, intracellular trafficking, downstream signaling networks, and associated changes in recipient-cell states. Disease microenvironmental variables, including cytokines, extracellular matrix composition, immune context, and vascular transport, could also be incorporated to support early-stage virtual pharmacodynamic assessment and potentially predict therapeutic responses across molecular, cellular, and microenvironmental scales. Created in BioRender. EV: Extracellular vesicle; ECM: extracellular matrix.

### Conceptual framework of digital twins in EV research

Digital twins are dynamic computational counterparts of biological systems that continuously integrate structural, molecular, and functional information to reproduce and predict real-world biological behavior^[16]^. Unlike static models, digital twins are adaptive systems that can be updated as new data become available. This dynamic characteristic is particularly important in EV research, where vesicle-mediated communication is highly sensitive to temporal changes in cellular state and tissue context. In EV therapeutics, digital twins could be developed at multiple biological scales. At the cellular level, they may model receptor expression, membrane transport, intracellular signaling, and metabolic status in recipient cells. At the tissue level, they can incorporate extracellular matrix composition, cytokine gradients, vascular transport, oxygen distribution, and immune cell infiltration. More advanced models could further integrate organ-level parameters such as circulation kinetics, tissue permeability, and systemic inflammatory status.

The scope of digital twins in EV biology therefore extends well beyond simulating vesicle uptake. By incorporating multi-omics datasets, imaging-derived spatial information, single-cell transcriptomic profiles, and prior biological knowledge, these systems could capture the dynamic interplay between EV characteristics and recipient cell responses. This may support a transition from empirical observation toward mechanistic prediction, allowing downstream biological consequences of EV administration to be estimated computationally in advance. Such predictive capacity is particularly valuable for engineered EVs, whose therapeutic performance depends on the coordinated effects of cargo composition, targeting ligands, membrane properties, vesicle size, and loading efficiency. Digital twins could therefore function not only as analytical tools, but also as decision-support systems for rational EV optimization.

One important advantage of digital twin frameworks is their capacity to integrate biological processes across multiple spatial and temporal scales. EV biology inherently spans nanoscale molecular recognition, cellular internalization, and tissue-level therapeutic responses. For example, ligand-receptor binding occurs at the molecular scale, whereas tissue penetration and biodistribution are influenced by organ-level transport processes. Digital twin frameworks address this complexity through hierarchical modeling architectures. Molecular simulations may incorporate ligand-receptor binding kinetics, membrane fusion energetics, or RNA-protein interactions. Cellular modules can model endocytosis, endosomal maturation, intracellular trafficking, and signaling activation. Tissue-level simulations then integrate interstitial diffusion, extracellular matrix density, vascular transport, and inflammatory microenvironmental conditions^[38]^.

Temporal integration is equally important. EV-mediated signaling may unfold over timescales ranging from seconds during membrane interactions to hours or days during transcriptional reprogramming and phenotypic remodeling. By incorporating time-dependent dynamics, digital twins could simulate the temporal evolution of EV behavior rather than represent it as isolated biological snapshots. This multiscale integration may improve the physiological relevance of computational predictions and enable examination of how local molecular events propagate into broader therapeutic outcomes^[64]^.

### Modeling EV uptake and intracellular trafficking

The initial stage of EV therapeutic activity involves interactions between vesicles and recipient-cell membranes. EV uptake is influenced by multiple factors, including surface ligands, receptor availability, extracellular matrix composition, and local inflammatory conditions^[57]^. Digital twin models may help estimate how these variables influence targeting selectivity and internalization efficiency under different biological contexts. Nevertheless, accurate modeling of EV uptake remains difficult because vesicle populations are highly heterogeneous and experimental measurements of internalization pathways are often inconsistent across studies. Differences in purification methods, labeling strategies, and imaging approaches can substantially alter the interpretation of uptake behavior. Together, these sources of variability make EV uptake difficult to model and may prevent simplified computational representations from fully capturing *in vivo* transport dynamics.

Following cellular internalization, EVs undergo intracellular trafficking processes that influence cargo stability and functional delivery^[57]^. Computational models could incorporate these processes to estimate how vesicle properties may affect endosomal processing, lysosomal degradation, and cytosolic cargo release. However, these processes remain incompletely understood experimentally, particularly at single-vesicle resolution. Cargo fate can vary considerably across recipient-cell types and microenvironmental conditions. This variability may limit the predictive precision of current EV-specific digital models.

### Prediction of downstream signaling and cellular responses

Following cytosolic release, EV-derived cargo components engage endogenous signaling and metabolic networks that collectively reshape recipient-cell behavior. These interactions are highly complex and involve pathway cross-talk, feedback regulation, compensatory signaling, and context-dependent responses^[65]^. Reductionist models centered on isolated pathways are therefore often insufficient for predicting EV-mediated effects. Digital twin systems could address this limitation by integrating signaling cascades, transcriptional regulatory networks, and metabolic pathways into unified computational frameworks. Such models could simulate how EV-derived miRNAs, proteins, metabolites, and lipids alter intracellular signaling dynamics over time^[58]^.

For example, EV cargo-associated miRNAs may suppress inflammatory signaling while promoting regenerative responses through specific molecular targets^[66]^. Transferred EV proteins can also modulate intracellular signaling and metabolic pathways^[58]^. Digital twin frameworks could therefore evaluate how multiple EV-derived signals reshape recipient-cell behavior under complex biological conditions. Such systems-level modeling is particularly relevant for EV therapeutics, whose biological activity may arise from coordinated interactions among multiple cargo components rather than isolated molecular effects. Through computational simulation, researchers could evaluate how different cargo architectures reshape global signaling landscapes in target cells.

Beyond intracellular signaling, digital twin frameworks could link molecular changes to higher-order phenotypic outcomes informed by experimentally observed EV effects^[67,68]^. By linking signaling dynamics to cellular functions, such models may help predict how EV treatment influences proliferation, differentiation, apoptosis, immune activation, senescence, extracellular matrix synthesis, or inflammatory responses. This capability is particularly valuable in complex pathological conditions involving interacting cell populations and heterogeneous tissue environments. In inflammatory diseases, for example, EV therapeutic responses depend not only on a single target cell type, but also on broader interactions among immune, stromal, vascular, and matrix components.

Digital twin simulations could allow systematic evaluation of these complex scenarios. Researchers could compare alternative EV designs under different pathological conditions and identify configurations that maximize therapeutic benefit while minimizing unintended effects. Such predictive modeling could reduce dependence on large-scale empirical screening and improve the efficiency of preclinical optimization.

### Microenvironment-aware modeling of EV function

The biological activity of EVs is strongly influenced by local microenvironmental conditions, including inflammatory status, extracellular matrix composition, metabolic state, and immune-cell interactions. These variables can substantially alter vesicle uptake, intracellular trafficking, and downstream signaling behavior^[69,70]^. Digital twin frameworks may therefore help incorporate disease-relevant biological contexts into computational models^[16]^.

An important advantage of microenvironment-aware modeling is the ability to analyze context-dependent therapeutic variability. EVs that show strong efficacy under one biological condition may display altered biodistribution, uptake efficiency, or signaling activity under another. Digital twins could enable systematic comparisons of EV behavior across different pathological contexts, patient-specific conditions, or disease stages. Such context-aware modeling may be particularly relevant to precision medicine because patient-specific microenvironmental differences can substantially influence EV therapeutic responses. By simulating these variations computationally, digital twins may support the development of personalized EV therapies optimized for specific biological conditions.

### Toward virtual pharmacodynamics of EVs

Digital twin systems may eventually support more quantitative evaluation of EV therapeutic behavior by integrating vesicle design features with biological response data^[16,23]^. Potential applications include estimation of therapeutic durability, tissue selectivity, and adverse biological responses under different pathological conditions. However, reliable pharmacodynamic prediction remains limited by incomplete mechanistic understanding and the lack of large-scale longitudinal datasets linking EV composition to functional outcomes.

Despite these limitations, digital twin frameworks may still help prioritize experimental strategies and refine EV engineering decisions. Through iterative integration of computational prediction and biological validation, these systems could gradually improve the efficiency and reproducibility of EV therapeutic development^[23]^.

## TOWARD CLOSED-LOOP AI-DRIVEN EV ENGINEERING

The integration of AIVCs, AI-guided EV design, and digital twin-based pharmacological modeling provides a conceptual basis for a prospective closed-loop paradigm in EV engineering^[23]^. Rather than proceeding through a linear sequence of modification, isolation, and validation, this emerging framework links design, prediction, testing, and refinement within a continuous adaptive cycle. By connecting computational models with experimental feedback, such closed-loop systems could provide a more systematic approach for addressing the biological complexity, heterogeneity, and manufacturing variability that have long limited EV translation.

Traditional EV engineering generally follows a sequential workflow in which parental cells are modified, vesicles are isolated, and biological activity is then assessed through *in vitro* or *in vivo* experiments. Although this strategy has generated valuable mechanistic insights, it remains constrained by several limitations. Experimental optimization is time-consuming and resource-intensive, only a limited portion of the design space can be explored empirically, and biological responses often depend on nonlinear interactions among multiple variables. Under these conditions, isolated parameter tuning is rarely sufficient to achieve robust improvements in therapeutic performance, driving interest in more adaptive design strategies.

Closed-loop AI-driven EV engineering could address these limitations by combining predictive computation with iterative experimental refinement. Within this framework, computational models generate candidate designs, experimental systems evaluate biological performance, and the resulting data are fed back into the models to refine subsequent predictions [Figure 5]. Over successive cycles, such systems could improve as experimentally validated data accumulate, representing a prospective shift from empirical trial-and-error toward predictive and systems-level EV design.

**Figure 5:**
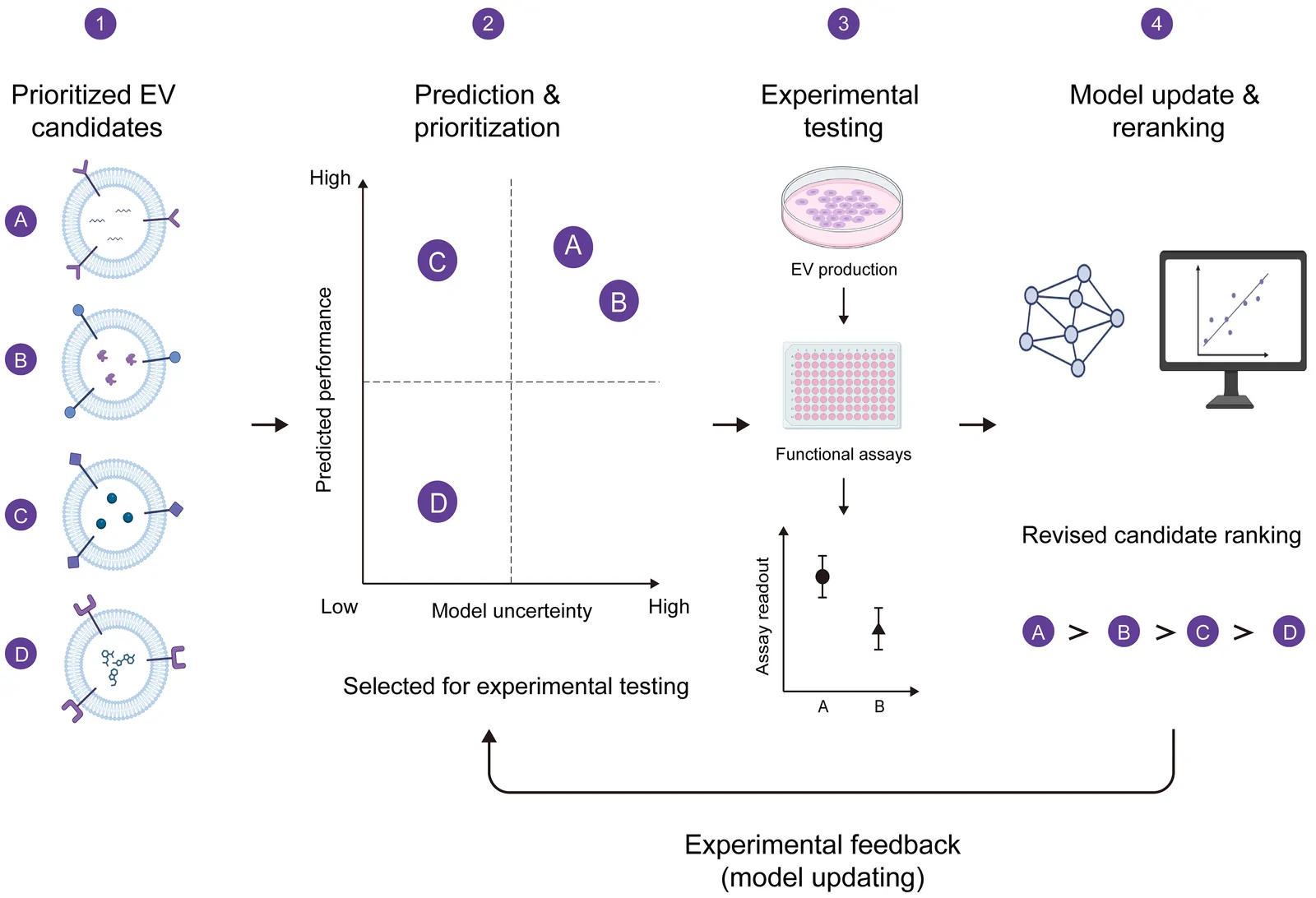
Closed-loop AI-driven EV engineering pipeline. The diagram presents an adaptive optimization cycle for EV therapeutic development. Prioritized EV candidates are first evaluated through computational prediction and prioritization, in which predicted performance and model uncertainty may help identify candidates for experimental testing. Selected vesicles are then produced and assessed through functional assays, and the resulting readouts can update the model and rerank candidates. Experimental feedback can subsequently inform model refinement, revised candidate ranking, and the selection of promising EV candidates for subsequent testing cycles. Created in BioRender. AI: Artificial intelligence; EV: extracellular vesicle.

### Architecture of the closed-loop EV engineering framework

Closed-loop AI-driven EV engineering could establish continuous interaction between computational prediction and experimental validation. Within this framework, AIVCs could model how parental-cell states influence EV biogenesis and cargo composition, AI-guided engineering systems may generate candidate vesicle designs, and digital twin models could simulate their downstream behavior^[23]^. Experimental systems would then provide empirical validation and biological feedback for subsequent optimization cycles. Rather than functioning as isolated stages, these components would operate as interconnected layers within an adaptive optimization pipeline. Such integration may reduce empirical screening burden and support more reproducible EV engineering strategies.

### Data integration and knowledge abstraction

The performance of closed-loop EV engineering systems depends on integrating heterogeneous datasets across multiple organizational scales. EV biology involves interactions at molecular, cellular, tissue, and systemic levels, so predictive modeling must account for these interconnected layers rather than focusing on a single dimension. Relevant data sources include molecular profiles of parental cells, EV cargo composition, membrane proteomics, lipidomics, intracellular signaling responses, microenvironmental characteristics, pharmacokinetic behavior, and therapeutic outcomes^[71]^. Temporal data are also important because EV-mediated responses evolve over time rather than remaining static.

AI-based frameworks can integrate these heterogeneous datasets into unified computational representations. Deep-learning architectures, including transformer- and graph-based models, can help integrate heterogeneous biological data and uncover latent relationships that are difficult to capture with conventional statistical approaches^[9,12,30,52]^. Alterations in parental-cell metabolism can reshape vesicle lipid organization, membrane biophysics, intracellular trafficking behavior, and downstream therapeutic activity simultaneously. Closed-loop architectures could capture these cross-scale dependencies within unified computational representations, potentially enabling systems-level optimization of EV function rather than isolated parameter adjustment.

A potential advantage of closed-loop systems is their ability to extract predictive patterns from accumulating experimental data. Rather than merely storing experimental outcomes, these systems may identify recurring relationships between EV design features and biological responses. As additional validated data become available, AI models may be updated and improve predictive performance. However, generalization across disease contexts should not be assumed and requires explicit external validation.

As larger and more standardized datasets become available, closed-loop frameworks may gradually improve their ability to support EV prediction, experimental prioritization, and therapeutic optimization across different biological settings. Such adaptive systems are especially relevant to regenerative medicine and nanotherapeutics, where incomplete mechanistic understanding and experimental variability substantially complicate therapeutic development. As these frameworks mature, they may support not only prediction but also experimental prioritization and strategy selection.

### Autonomous optimization and iterative design

EV therapeutics occupy a highly complex design space involving vesicle size, membrane composition, cargo architecture, targeting ligands, parental-cell state, and manufacturing conditions^[7,49]^. Because these variables interact nonlinearly, exhaustive empirical optimization is often impractical. AI-driven systems may therefore efficiently prioritize vesicle configurations with favorable predicted therapeutic behavior before experimental validation.

Experimental validation nevertheless remains essential because biological systems frequently display context-dependent behaviors that cannot be fully captured computationally. Discrepancies between predicted and observed outcomes could subsequently be reintegrated into the computational framework to improve model robustness and predictive accuracy. Over successive refinement cycles, this adaptive process may support progressively more reproducible EV designs, although predicted therapeutic improvements still require experimental confirmation.

### Toward autonomous EV research and development systems

Integration of AI-guided design with automated experimental platforms may further improve the efficiency and reproducibility of EV research. Technologies established in adjacent fields, including robotic experimentation and self-driving laboratory systems, demonstrate how automated testing can be coupled with computational design and iterative feedback^[25]^. Such approaches could be adapted to EV production, screening, imaging, and data acquisition. In parallel, active-learning frameworks may assist in prioritizing experiments that provide high informational value for model refinement^[39]^. Although fully autonomous EV development systems remain unrealistic at present, partial automation may accelerate optimization workflows and improve translational scalability.

### Personalized and precision EV therapeutics

Closed-loop EV engineering could provide a framework for precision nanomedicine^[27]^. Because disease progression, immune responses, and tissue microenvironments vary substantially among individuals, standardized EV formulations may not produce equivalent therapeutic effects across all patients. By incorporating patient-specific biological information, including genomic profiles, transcriptomic signatures, inflammatory biomarkers, imaging data, and disease characteristics, closed-loop frameworks could in principle support individualized response modeling^[16,27]^. Digital twin models may then help estimate how these variables influence EV biodistribution, uptake behavior, intracellular signaling, and therapeutic responses. Such modeling could support the computational optimization of EV formulations for different biological contexts.

Closed-loop personalization could be envisioned as a dynamically adaptive process in which evolving patient-derived biological data inform EV design parameters throughout disease progression and therapeutic intervention. These parameters could therefore be iteratively adjusted based on disease progression, treatment response, or evolving inflammatory conditions. In chronic degenerative diseases, the tissue microenvironment may shift during treatment, and future closed-loop systems could adapt EV cargo or targeting strategies to these changing conditions. Such adaptive capacity would represent a transition from generalized treatment strategies toward more responsive precision therapeutics. If successfully translated to clinical settings, this approach could allow EV engineering to evolve toward a longitudinal rather than single-event design process.

### System-level integration and translational significance

The operational value of closed-loop AI-driven EV engineering lies in its ability to connect design, prediction, validation, and refinement within a continuous workflow. In this system, computational models do not simply generate static EV designs; instead, they function as decision-support tools that prioritize candidate vesicle configurations for experimental testing. Experimental platforms then evaluate key biological outputs, including EV yield, cargo composition, targeting efficiency, cellular uptake, functional potency, and safety-related features. The resulting data are reintegrated into the computational models to update design parameters and improve subsequent prediction accuracy.

This iterative process could transform EV engineering from a one-directional experimental workflow into an adaptive optimization system. Each cycle could refine different levels of the therapeutic design, including parental-cell conditioning, cargo selection, surface architecture, vesicle stability, or disease-specific functional performance. As the feedback dataset expands, the system may better identify which design features are reproducibly associated with favorable biological outcomes. In this way, closed-loop engineering provides a testable framework to reduce empirical trial-and-error, improve reproducibility, and prioritize EV candidates for further validation.

Nevertheless, this framework's effectiveness depends critically on the quality and standardization of input data, the reliability of computational models, and the biological relevance of validation systems. Closed-loop EV engineering should therefore be understood as an adaptive development strategy rather than a fully autonomous therapeutic platform. Its current value lies in organizing complex EV design variables into a testable and iterative workflow, while broader implementation will require solutions to the technical and translational barriers discussed in the following section.

## CHALLENGES AND FUTURE DIRECTIONS

Despite the conceptual promise of AI-guided EV engineering, its practical implementation remains limited by several unresolved barriers. The main challenges are no longer simply whether computational models can be introduced into EV research, but whether these models can be trained on reliable datasets, generate biologically interpretable predictions, operate across multiple biological scales, and be validated under clinically relevant conditions. At present, AI-enabled EV workflows remain largely early-stage research frameworks rather than mature translational platforms^[23]^.

These limitations arise from the intrinsic complexity of EV biology. EV populations are heterogeneous, dynamically regulated by parental-cell states, and highly sensitive to isolation, purification, culture, storage, and characterization procedures. As a result, computational predictions may be strongly influenced by technical variability, incomplete biological annotation, and insufficient linkage between EV composition and functional outcomes. In addition, many current models focus on isolated aspects of EV behavior, such as cargo prediction, uptake, or biodistribution, whereas therapeutic efficacy depends on coordinated interactions among vesicle properties, recipient-cell states, tissue microenvironments, and systemic physiology.

Therefore, the future development of AI-driven EV therapeutics will depend on progress in four major areas: standardized and functionally annotated EV datasets, interpretable and biologically constrained AI models, multiscale digital twin systems that better reflect disease microenvironments, and translational validation pipelines compatible with manufacturing and regulatory requirements. Figure 6 provides an overview of selected challenges and potential directions for the further development and clinical translation of AI-enabled EV engineering. The following sections discuss these barriers and outline how they may be addressed to move AI-guided EV engineering from conceptual design toward reproducible and clinically relevant therapeutic development.

**Figure 6:**
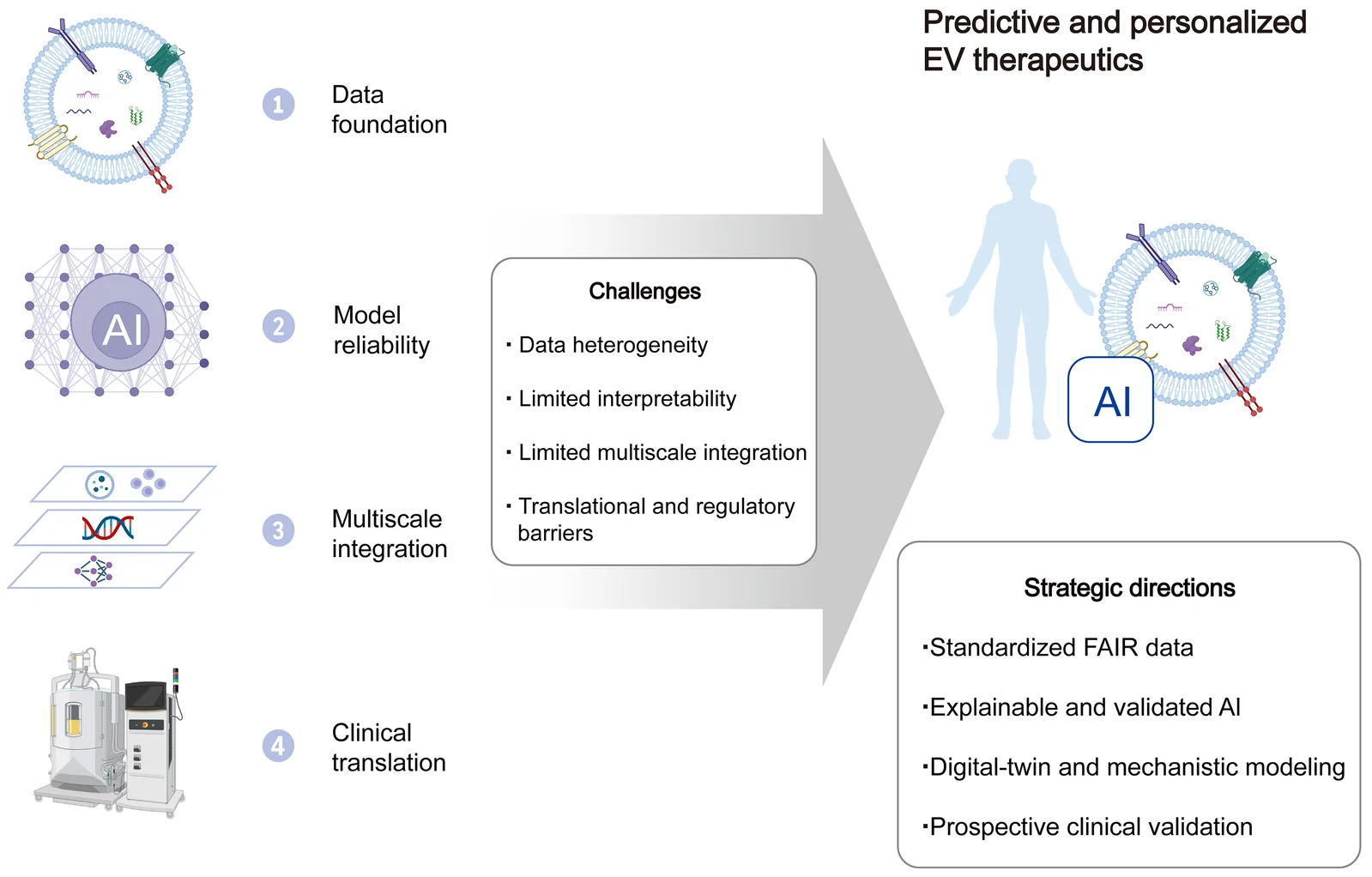
Challenges and roadmap for AI-driven EV engineering. The roadmap summarizes major barriers and future directions for AI-enabled EV therapeutics. Current limitations include EV data heterogeneity, limited interpretability, insufficient multiscale integration, and translational or regulatory uncertainty. Future progress may depend on standardized, findable, accessible, interoperable, and reusable (FAIR) data, explainable and validated AI, digital-twin and mechanistic modeling, and prospective clinical validation. These advances may support the gradual transition of AI-driven EV engineering from early conceptual frameworks toward more predictive, personalized, and translationally feasible EV nanomedicine. Created in BioRender. AI: Artificial intelligence; EV: extracellular vesicle; FAIR: findable, accessible, interoperable, and reusable.

### Data limitations and standardization

A foundational prerequisite for AI-driven EV engineering is the availability of high-quality, standardized, and functionally annotated datasets. Although omics technologies have rapidly expanded transcriptomic, proteomic, metabolomic, and epigenomic profiling at the cellular level, EV-specific datasets remain comparatively underdeveloped, particularly those capable of resolving vesicle heterogeneity with high spatial and molecular precision^[26,29]^.

This challenge largely originates from the intrinsic biological complexity of EV populations. EV preparations isolated from biological samples frequently contain heterogeneous EV subtypes together with co-isolated non-vesicular components, including protein aggregates and lipoprotein-associated particles. Even vesicles secreted by the same parental cell population may differ substantially in size, membrane composition, cargo architecture, and biological activity. Conventional bulk characterization approaches further obscure this complexity by averaging signals across diverse vesicle populations, thereby masking functionally distinct EV subtypes and limiting identification of biologically meaningful relationships^[26,48]^.

Insufficient biological resolution poses a major constraint on AI model development. Machine learning frameworks rely on accurate associations between molecular features and biological outputs, yet bulk datasets often conceal critical determinants governing EV function, cargo loading efficiency, biodistribution, and therapeutic activity. Predictive accuracy and mechanistic interpretability remain limited. Addressing these issues will require broader adoption of high-resolution analytical approaches, including nanoscale flow cytometry, super-resolution imaging, single-vesicle sequencing, and advanced spatial omics platforms. Such approaches may provide a more refined understanding of EV subpopulations and may improve the biological fidelity of AI training datasets.

Technical inconsistency across current experimental workflows further complicates data integration. Existing EV isolation, purification, characterization, and quantification strategies vary considerably among laboratories. Different ultracentrifugation protocols, precipitation methods, filtration systems, chromatographic approaches, and microfluidic platforms enrich distinct EV populations and introduce method-specific biases. Variability in sample preparation, storage conditions, sequencing pipelines, normalization procedures, and functional assays further reduces comparability across studies, making the integration of independent datasets into unified computational frameworks particularly difficult.

The consequences of this variability extend beyond technical inconsistency. Training datasets may capture methodological artifacts rather than authentic biological relationships, thereby compromising reproducibility and reducing model robustness. In addition, the absence of harmonized experimental standards limits external validation across independent cohorts and restricts scalability of collaborative multi-center studies necessary for large-scale AI development. Establishment of internationally harmonized standards for EV isolation, characterization, reporting, and data annotation will be essential for improving dataset interoperability and large-scale model development^[26,48]^.

Another major challenge is the limited availability of datasets directly linking EV composition to downstream functional outcomes^[29,71]^. Many currently available datasets primarily describe molecular cargo profiles without adequately integrating pharmacokinetic behavior, biological responses, or therapeutic efficacy under defined experimental conditions. However, predictive EV engineering ultimately requires computational models capable of connecting vesicle composition with functional phenotypes across complex biological contexts.

Temporal resolution also remains insufficient in most existing datasets. EV-mediated biological responses are highly dynamic processes involving sequential alterations in cellular uptake, intracellular signaling, immune regulation, and tissue remodeling. Because most currently available datasets are cross-sectional rather than longitudinal, important temporal relationships governing EV pharmacology often remain unresolved. Continued progress will therefore require not only larger datasets, but also integrative experimental systems that can simultaneously capture EV composition, spatial distribution, temporal evolution, and functional biological outcomes within unified analytical frameworks.

### Model interpretability and reliability

As AI models become increasingly important in EV engineering, ensuring their interpretability and reliability is critical. Many advanced machine learning models, particularly deep neural networks and transformer-based architectures, operate as highly complex nonlinear systems capable of generating accurate predictions while offering limited mechanistic transparency. In the context of EV therapeutics, predictive accuracy alone is insufficient. EV-based interventions involve direct interactions with highly sensitive biological systems, and therefore both safety and mechanistic understanding are essential. Clinicians, researchers, and regulatory agencies must be able to understand how a computational system arrives at a specific design recommendation or therapeutic prediction. The “black-box” nature of many AI models presents significant challenges in this regard^[72]^. Without interpretability, it becomes difficult to determine whether a prediction reflects biologically meaningful relationships or merely statistical correlations within the training dataset. This uncertainty limits confidence in computationally generated EV designs, particularly in translational and clinical contexts.

Improving interpretability requires incorporation of explainable AI methodologies capable of identifying which biological features contribute most strongly to model predictions. Attention mechanisms, feature attribution analyses, causal inference frameworks, and biologically constrained architectures may help reveal the molecular logic underlying computational outputs. Equally important is the integration of prior biological knowledge directly into model design. Rather than relying solely on data-driven learning, AI systems can incorporate known signaling pathways, vesicle trafficking mechanisms, receptor-ligand interactions, and intracellular regulatory networks. This hybrid strategy may improve both mechanistic interpretability and biological plausibility. For example, GNNs structured according to experimentally validated molecular interaction networks may provide more biologically meaningful predictions than unconstrained deep learning systems. Similarly, mechanistic pathway modeling combined with probabilistic inference may improve generalizability across different biological conditions.

Rigorous experimental validation is essential for establishing confidence in AI-generated predictions. Computational outputs must be systematically compared with biological observations across multiple independent experimental systems, including *in vitro* cultures, organoids, microphysiological systems, and animal models. Standardized benchmarking frameworks will therefore become increasingly important. Such frameworks should include reproducibility metrics, predictive-accuracy criteria, robustness assessments, and external-validation standards. Importantly, benchmarking systems must evaluate not only predictive performance but also biological interpretability and translational reliability.

### Multiscale integration and biological complexity

EV biology spans multiple interconnected biological scales, ranging from nanoscale molecular interactions to tissue-level therapeutic responses. Cargo sorting involves molecular recognition events among RNAs, proteins, lipids, and intracellular trafficking machinery. Vesicle uptake depends on membrane biophysics, receptor expression, and extracellular matrix interactions. Downstream therapeutic efficacy further depends on tissue architecture, immune modulation, vascular transport, and systemic physiological conditions^[56,57]^. Capturing this complexity within unified computational systems remains a major scientific challenge. Current AI models often focus on isolated aspects of EV behavior, such as cargo prediction, biodistribution, or uptake dynamics. However, biological systems operate through extensive cross-scale interactions that isolated modeling approaches cannot fully capture.

Digital twin technologies may provide a promising strategy for multiscale integration because they could support the simultaneous modeling of molecular, cellular, tissue, and physiological processes. Nevertheless, implementing such systems requires coordination of highly heterogeneous data types and computational frameworks. For example, molecular interaction models may rely on graph-based network architectures, whereas tissue-level transport simulations may use systems of differential equations or agent-based modeling. Integrating these distinct computational approaches into interoperable platforms remains technically demanding. Modular computational infrastructures that link independent modeling layers while preserving computational efficiency may be important for future multiscale EV engineering systems. Such architectures must also remain sufficiently flexible to incorporate newly emerging biological datasets and experimental technologies.

Another major consideration is computational scalability. High-resolution simulations of EV behavior across multiple biological scales require substantial computational resources, particularly when incorporating temporal dynamics and stochastic biological variability. Balancing biological realism with computational tractability therefore remains a critical optimization problem. Excessively simplified models may fail to capture relevant biological processes, whereas overly detailed systems may become computationally impractical for iterative optimization workflows. Advances in cloud computing, distributed AI systems, high-performance computational infrastructure, and model compression strategies may help address these challenges in future large-scale EV engineering platforms.

### Translational and regulatory considerations

The translation of AI-engineered EVs into clinical applications introduces additional challenges related to manufacturing reproducibility, quality control, and biosafety. Unlike conventional small-molecule therapeutics, EV-based products are biologically complex and highly sensitive to production conditions^[49,73]^. Minor alterations in parental cell state, culture environment, nutrient availability, oxygen tension, or isolation methodology may substantially alter EV composition and therapeutic behavior. AI-guided engineering further increases system complexity by introducing computationally optimized vesicle architectures that may be difficult to standardize experimentally. Ensuring reproducible large-scale manufacturing will therefore require highly controlled production systems, automated quality monitoring, and standardized release criteria.

Integrating adaptive AI with EV-based biologic products may raise regulatory questions that existing frameworks do not fully address. Additional guidance may be needed to address issues such as algorithm validation, model traceability, computational transparency, and continuous model updating^[74,75]^. Regulatory agencies may need to evaluate not only the final EV product but also the computational processes used during design and optimization. This introduces new questions regarding acceptable model uncertainty, reproducibility of AI-generated designs, and long-term system stability. Collaboration among computational scientists, clinicians, bioengineers, and regulatory organizations will therefore be essential for establishing practical translational pathways for AI-driven EV therapeutics.

### Future perspectives: toward predictive and personalized EV therapeutics

Despite these challenges, continued advances in data acquisition, computational biology, and automated experimentation may accelerate the evolution of AI-driven EV engineering. Increasing availability of high-resolution multi-omics datasets, combined with improvements in machine learning architectures and causal inference systems, may enhance predictive accuracy and biological interpretability. At the same time, maturation of digital twin technologies may enable increasingly realistic simulation of EV behavior within complex biological systems. Integration of these frameworks with patient-specific molecular and clinical data may support highly personalized therapeutic strategies.

One promising future direction is developing individualized EV therapeutics tailored to patient-specific biological conditions. By incorporating genomic, transcriptomic, immunological, and pathological information into closed-loop computational systems, AI frameworks may predict which EV configurations best suit individual patients. These systems may also support adaptive therapeutic optimization. As patient responses evolve over time, updated biological data can be reintegrated into the computational framework, allowing iterative adjustment of EV design parameters according to disease progression or therapeutic response. Such adaptive personalization represents a significant conceptual transition from generalized nanotherapeutics toward continuously optimized precision medicine.

In parallel, advances in laboratory automation, robotic experimentation, and AI-assisted experimental planning may enable partially autonomous EV engineering platforms. These systems could continuously generate hypotheses, perform experimental validation, analyze biological outputs, and refine computational models with minimal human intervention. By enabling predictive, adaptive, and personalized design strategies, AI-driven EV engineering may provide a pathway toward more precise, scalable, and biologically informed therapeutic systems. Although substantial scientific and translational challenges remain, ongoing interdisciplinary progress may gradually advance this field from an emerging conceptual framework toward clinically relevant applications in next-generation medicine.

## CONCLUSION

AI is increasingly being explored to make EV engineering more predictive and less dependent on empirical trial-and-error. Conventional EV engineering has been constrained by biological heterogeneity, limited targeting precision, variable cargo composition, and reliance on repeated empirical optimization. By integrating multi-omics data, computational modeling, digital twin simulation, and experimental feedback, AI-driven frameworks provide a conceptual route for addressing these limitations and support the rational design of EV therapeutics. Distinct from reviews that primarily discuss individual AI applications in EV analysis or engineering, this review organizes parental-cell programming, cargo and surface co-design, digital twin modeling, and experimental feedback within an evidence-aware, multiscale framework that distinguishes established EV evidence from transferable technologies and prospective concepts.

At the source level, AIVCs offer a framework for modeling how parental-cell states, intracellular perturbations, and biogenesis pathways may influence EV secretion and cargo composition. This perspective allows EVs to be considered not merely as biological byproducts, but as tunable outputs of dynamic cellular systems. At the vesicle level, AI-guided engineering may support coordinated design of therapeutic cargo networks, surface ligands, loading efficiency, and membrane architecture. These approaches could move EV design beyond single-component modification toward more integrated engineering of vesicle function. At the recipient-system level, digital twin technologies could complement EV design by simulating uptake, intracellular trafficking, microenvironmental interactions, and downstream pharmacodynamic responses. When combined with iterative experimental validation, these computational tools may help improve the efficiency and reproducibility of EV therapeutic development, although their translational value remains to be established.

Nevertheless, AI-driven EV engineering remains an emerging framework rather than a mature clinical platform. Its future progress will depend first on the availability of high-quality, standardized, and functionally annotated EV datasets, particularly those linking vesicle composition, single-vesicle heterogeneity, biodistribution, and therapeutic outcomes. Equally important is developing interpretable, biologically constrained AI models that generate mechanistically meaningful predictions rather than purely statistical associations. Multiscale integration also remains a major challenge, as EV therapeutic activity depends on coordinated interactions among parental-cell regulation, vesicle architecture, recipient-cell signaling, tissue microenvironments, and systemic physiology. In addition, scalable manufacturing, quality control, biosafety evaluation, and regulatory alignment should be incorporated into AI-guided workflows from the early stages of development.

Future advances in EV therapeutics may increasingly draw on the convergence of AI, systems biology, automated experimentation, and precision medicine. Rather than replacing biological validation, AI should function as a decision-support and optimization framework that prioritizes candidate designs, reduces the burden of empirical screening, and refines therapeutic strategies through iterative feedback. As high-resolution datasets, explainable modeling systems, digital twin platforms, and standardized manufacturing pipelines continue to mature, AI-driven EV engineering may gradually advance from a conceptual research paradigm toward more predictive and personalized therapeutic development. Its main value may therefore lie in helping researchers prioritize testable EV designs and refine them as new biological data become available.

## DECLARATIONS

### Copyright

© The Author(s) 2026.
